# Supplementary material for: Calorie restriction increases insulin sensitivity to promote beta cell homeostasis and longevity in mice
Source: Nat Commun. 2024 Oct 21;15:9063. doi: 10.1038/s41467-024-53127-2 (PMC11493975; doi:10.1038/s41467-024-53127-2)
Supplement: Supplementary file 1 — Supplementary Information [file 41467_2024_53127_MOESM1_ESM.pdf]

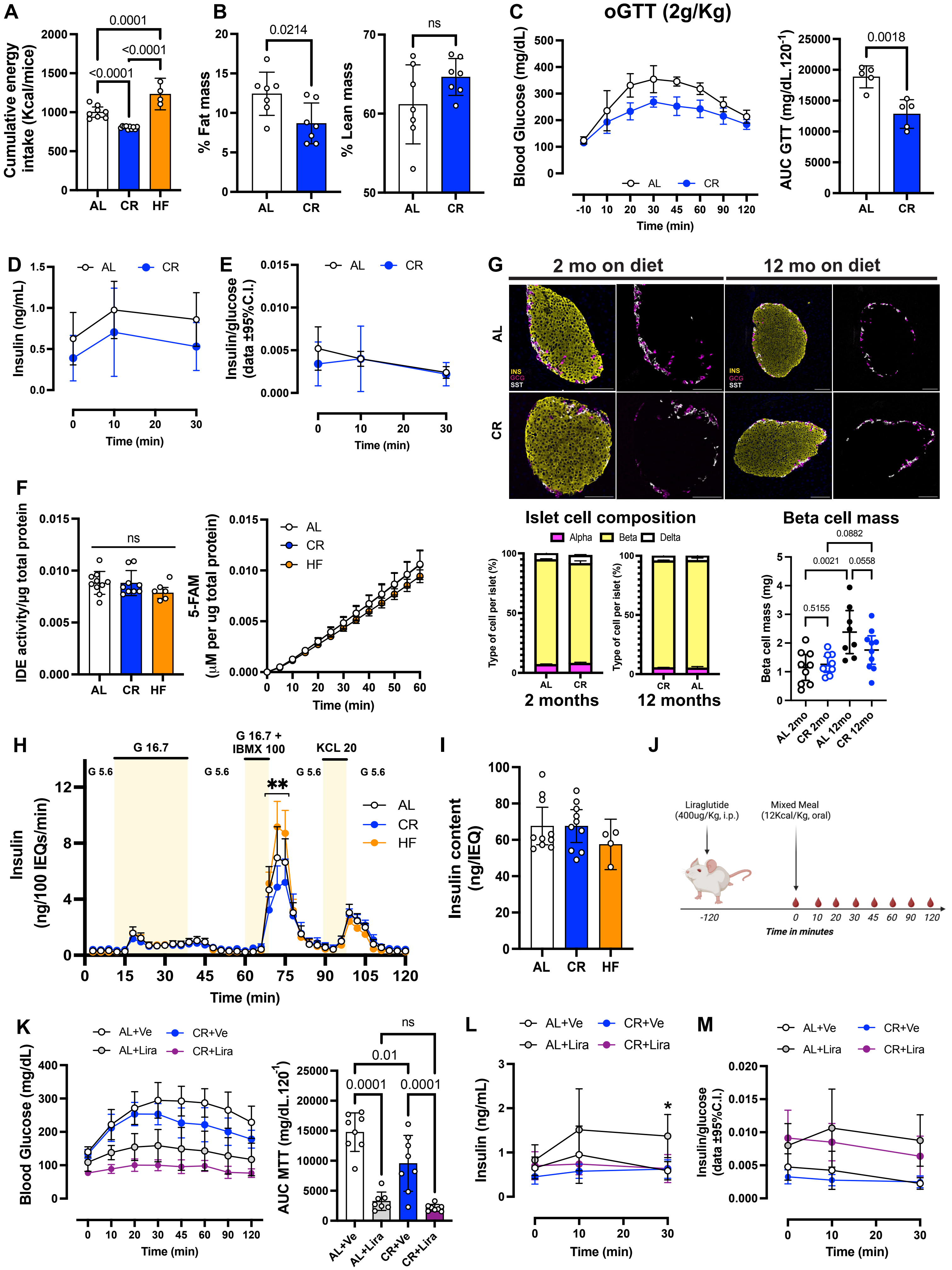
A
Cumulative energy intake (Kcal/mice)
0.0001
<0.0001
<0.0001
AL
CR
HF
B
% Fat mass
0.0214
% Lean mass
ns
C
oGTT (2g/Kg)
Blood Glucose (mg/dL)
Time (min)
AUC GTT (mg/dL.120^-1)
0.0018
D
Insulin (ng/mL)
Time (min)
E
Insulin/glucose (data ±95%C.I.)
Time (min)
G
2 mo on diet
12 mo on diet
INS
GCG
SST
F
IDE activity/µg total protein
ns
5-FAM (µM per ug total protein)
Time (min)
Islet cell composition
Alpha
Beta
Delta
Type of cell per islet (%)
2 months
12 months
Beta cell mass
Beta cell mass (mg)
0.0882
0.0021
0.0558
0.5155
AL 2mo
CR 2mo
AL 12mo
CR 12mo
H
G 16.7
G 16.7 + IBMX 100
KCL 20
G 5.6
**
Insulin (ng/100 IEQs/min)
Time (min)
I
Insulin content (ng/IEQ)
J
Liraglutide (400ug/Kg, i.p.)
Mixed Meal (12Kcal/Kg, oral)
-120
0
10
20
30
45
60
90
120
Time in minutes
K
AL+Ve
CR+Ve
AL+Lira
CR+Lira
Blood Glucose (mg/dL)
Time (min)
AUC MTT (mg/dL.120^-1)
ns
0.01
0.0001
0.0001
L
Insulin (ng/mL)
*
Time (min)
M
Insulin/glucose (data ±95%C.I.)
Time (min)

Supplementary Figure 1 - Related to Figure 1. (A) Cumulative energy intake over 2 months on diet from FVB male mice fed with AL, 20% CR or HFD. Each point represents the average from each cage with five mice, n=4-10 cages per experimental group from two different sets. **(B)** Percentage of fat and lean mass per body weight from AL and CR male mice. **(C)** Blood glucose levels during the oral glucose tolerance test (oGTT) after 2 months on diet and respective area-under-curve (AUC) measurements. **(D)** Insulin levels during the oGTT and **(E)** the respective ratio between the insulin and glucose values. **(F)** Insulin degrading enzyme activity measured in liver samples from male mice after 2 months on diet. **(G)** Pancreatic islet morphology from male mice after 2 or 12 months on diet. Beta, alpha and delta cells were detected by immunofluorescence, using anti-insulin (yellow), anti-glucagon (magenta) and anti-somatostatin antibodies (white). Quantitative analysis of beta-cell mass and the percentage of beta, alpha and delta-cells per islet were performed in two different sections from each pancreas. **(H)** Dynamic insulin secretion obtained from perifused islets with insulin secretagogues (5.6 mM and 16.7 mM glucose, IBMX 100 mM, and KCl 20mM) and the respective **(I)** insulin total content normalized by IEQ. **(J)** Schematic diagram of mice subjected to liraglutide (400 ug/Kg i.p) 2 hours before the MTT. **(K)** Blood glucose levels during the MTT using mice pretreated with liraglutide and respective AUC measurements. **(L)** Insulin levels during the MTT and **(M)** the respective ratio between the insulin and glucose values. In (B), n=7 male mice per diet group; (C-E) n=5 male mice per diet group; (F) n=6-9 male mice per diet group; (G) n=9-10 male mice per diet group; (H-I) n=5-10 male mice per diet group. (K-M) n=8 male mice per diet group. Statistical analysis was conducted using one-way or two-way ANOVA with Tukey's post-hoc test for multiple comparison (A, D-M), or unpaired two-tailed Student's t-test (B-C). In (H), the asterisks indicate ** $p < 0.01$. All data presented as mean ± 95% CI. Panels 1J was created with BioRender.com. and released under a Creative Commons Attribution-Non-Commercial NoDerivs 4.0 International license.

C57BL6J mice

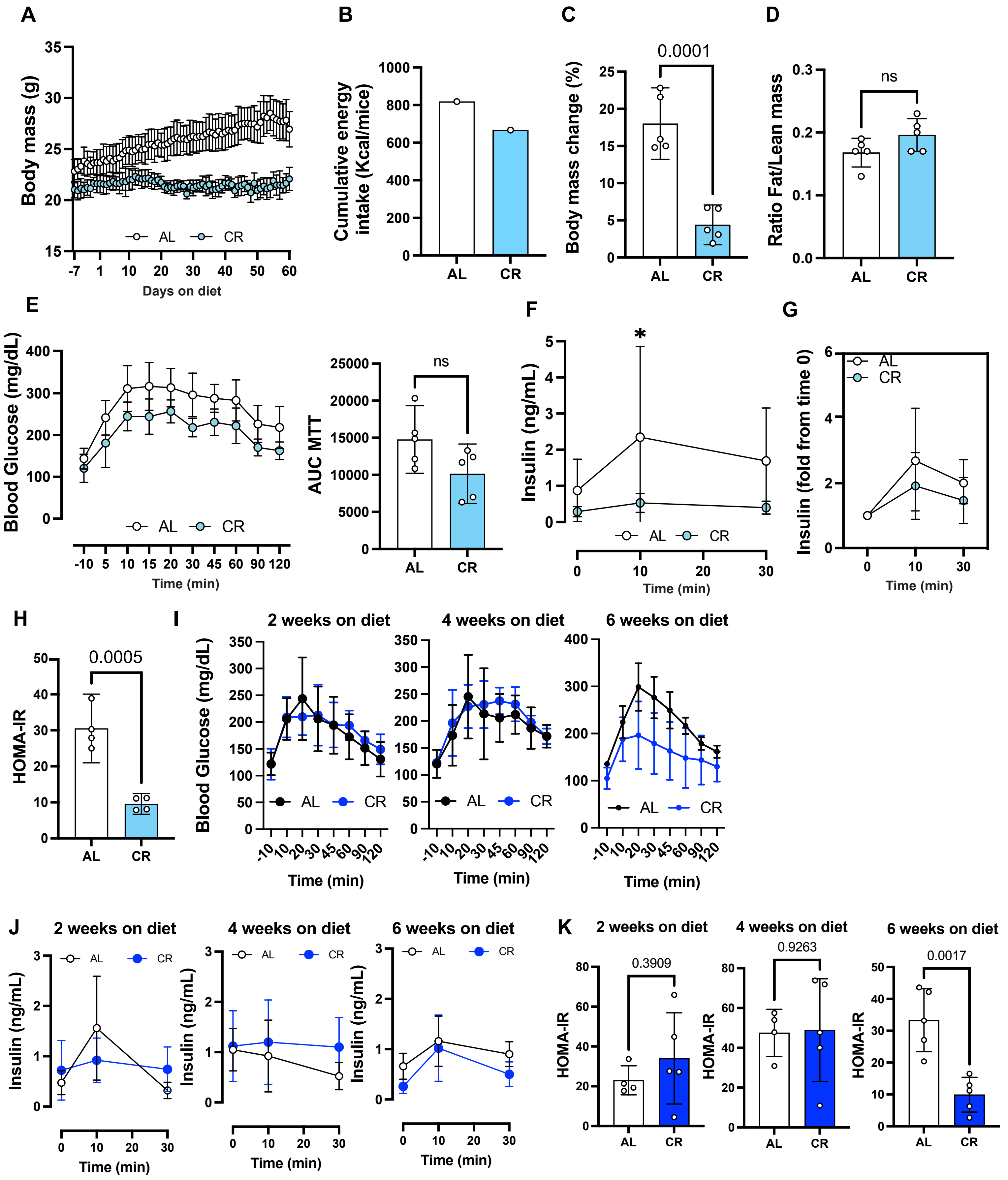

Supplementary Figure 2 – Related to Figure 1. (A) Daily body mass of C57BL6J male mice fed with AL or 20% CR for 2 months. **(B)** Cumulative energy intake over 2 months on diet, each point represents the average from each cage with five mice. **(C)** Body mass change and **(D)** the ratio of fat and lean mass. **(E)** Blood glucose levels obtained during the meal tolerance test (MTT) after 2 months on diet and the respective AUC. **(F)** Insulin levels obtained during the MTT and **(G)** the respective fold change from baseline values. **(H)** HOMA-IR calculated from fasting glucose and insulin values after 2 months on diet. **(I-K)** Blood glucose levels obtained during a meal tolerance test (MTT), circulating insulin levels during the MTT, and HOMA-IR index measured after 2-, 4-, or 6-weeks on CR diet. Statistical analysis was conducted using two-way ANOVA with Tukey's post-hoc test for multiple comparison (F-G, I-J), or unpaired two-tailed Student's t-test (C-E, H-K). In (F), the asterisks indicate * $p < 0.05$. n=5 male mice per diet group. All data presented as mean ± 95% CI.

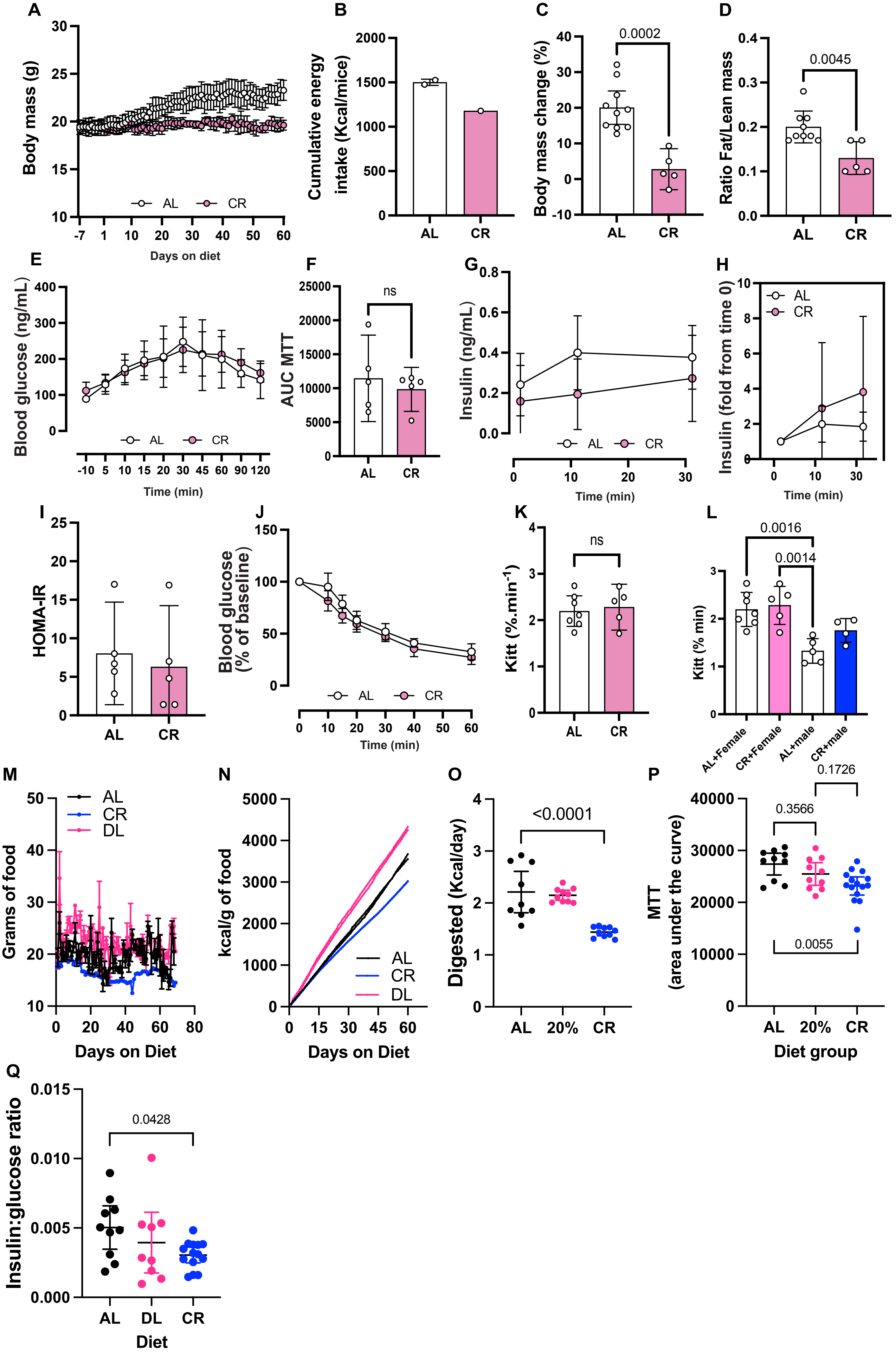
A
Body mass (g)
Days on diet
AL
CR
B
Cumulative energy intake (Kcal/mice)
AL
CR
C
Body mass change (%)
0.0002
AL
CR
D
Ratio Fat/Lean mass
0.0045
AL
CR
E
Blood glucose (ng/mL)
Time (min)
AL
CR
F
AUC MTT
ns
AL
CR
G
Insulin (ng/mL)
Time (min)
AL
CR
H
Insulin (fold from time 0)
Time (min)
AL
CR
I
HOMA-IR
AL
CR
J
Blood glucose (% of baseline)
Time (min)
AL
CR
K
Kitt (%.min-1)
ns
AL
CR
L
Kitt (% min)
0.0016
0.0014
AL+Female
CR+Female
AL+male
CR+male
M
Grams of food
Days on Diet
AL
CR
DL
N
kcal/g of food
Days on Diet
AL
CR
DL
O
Digested (Kcal/day)
<0.0001
AL
20%
CR
P
MTT (area under the curve)
0.3566
0.1726
0.0055
AL
20%
CR
Diet group
Q
Insulin:glucose ratio
0.0428
AL
DL
CR
Diet

Supplementary Figure 3 – Related to Figure 1. (A) Daily body mass of female mice fed with AL or 20% CR for 2 months. **(B)** Cumulative energy intake over 2 months on diet, each point represents the average from each cage with five mice, n=2-1 cages per experimental group. **(C)** Body mass change and **(D)** the ratio of fat and lean mass. **(E)** Blood glucose levels obtained during the meal tolerance test (MTT) and the respective AUC in **(F)**. **(G)** Insulin levels obtained during the MTT and **(H)** the respective fold change from baseline values. **(I)** HOMA-IR calculated from fasting glucose and insulin values after 2 months on diet. **(J)** Blood glucose levels obtained during the insulin tolerance test (ipITT) and **(K)** the respective decay of the glucose rate per minute (kITT). **(L)** Differences in kITT between male and female mice subject to AL or 20% CR for 2 months. n=5 female mice per diet group. **(M)** Daily food consumption of male mice placed on AL, CR, or DL diet for 2 months. **(N)** Cumulative energy intake of AL, CR, and DL mice. **(O)** Energy available from food digestion calculated by bomb calorimetry of fresh fecal matter collected from AL, CR, or DL mice. **(P-Q)** MTT test area under the curve and in insulin to glucose ratio, respectively, from AL, CR, or DL mice, n=9-15 mice per group (M-Q). Statistical analysis was conducted using one-way or two-way ANOVA with Tukey's post-hoc test for multiple comparison (G, H, L, O-Q), or unpaired two-tailed Student's t-test (C, D, F, I, K). All data presented as mean ± 95% CI.

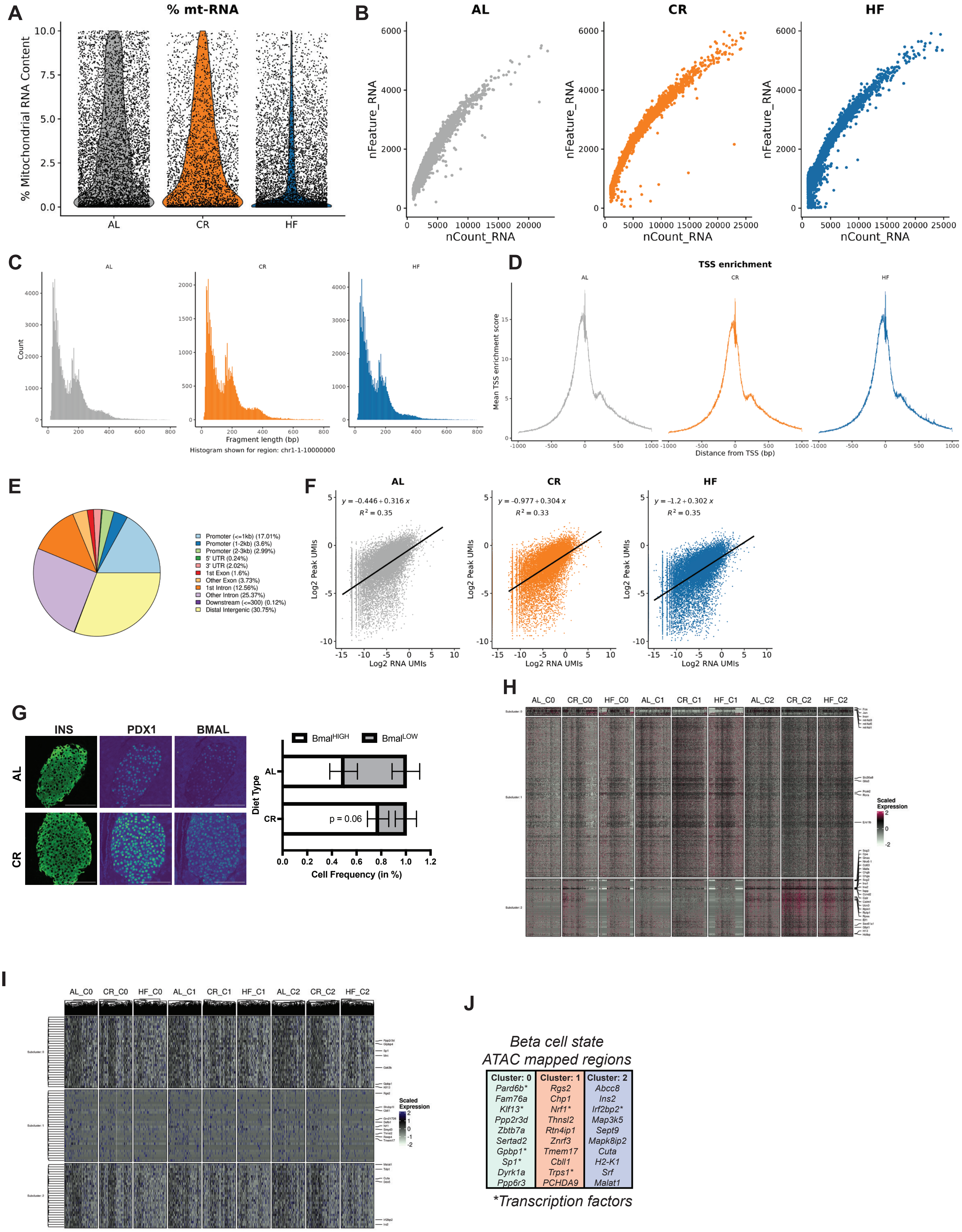
A
% mt-RNA
% Mitochondrial RNA Content
AL
CR
HF
B
AL
CR
HF
nFeature_RNA
nCount_RNA
C
AL
CR
HF
Count
Fragment length (bp)
Histogram shown for region: chr1-1-10000000
D
TSS enrichment
AL
CR
HF
Mean TSS enrichment score
Distance from TSS (bp)
E
Promoter (<=1kb) (17.01%)
Promoter (1-2kb) (3.6%)
Promoter (2-3kb) (2.99%)
5' UTR (0.24%)
3' UTR (2.02%)
1st Exon (1.6%)
Other Exon (3.73%)
1st Intron (12.56%)
Other Intron (25.37%)
Downstream (<=300) (0.12%)
Distal Intergenic (30.75%)
F
AL
CR
HF
y = -0.446 + 0.316 x
R² = 0.35
y = -0.977 + 0.304 x
R² = 0.33
y = -1.2 + 0.302 x
R² = 0.35
Log2 Peak UMIs
Log2 RNA UMIs
G
INS
PDX1
BMAL
AL
CR
Bmal HIGH
Bmal LOW
Diet Type
p = 0.06
Cell Frequency (in %)
H
AL_C0
CR_C0
HF_C0
AL_C1
CR_C1
HF_C1
AL_C2
CR_C2
HF_C2
Scaled Expression
I
AL_C0
CR_C0
HF_C0
AL_C1
CR_C1
HF_C1
AL_C2
CR_C2
HF_C2
Scaled Expression
J
Beta cell state
ATAC mapped regions
Cluster: 0
Pard6b*
Fam76a
Klf13*
Ppp2r3d
Zbtb7a
Sertad2
Gpbp1*
Sp1*
Dyrk1a
Ppp6r3
Cluster: 1
Rgs2
Chp1
Nrf1*
Thnsl2
Rtn4ip1
Znrf3
Tmem17
Cbll1
Trps1*
PCHDA9
Cluster: 2
Abcc8
Ins2
Irf2bp2*
Map3k5
Sept9
Mapk8ip2
Cuta
H2-K1
Srf
Malat1
*Transcription factors

Supplementary Figure 4. Related to Figure 3. **(A)** Fraction of mitochondrial (mt) genes expressed in all sequenced cells from AL, CR, and HFD mice. **(B)** Graph with number of genes (features) identified as a function of the number of RNA molecules detected (nCount) in cells from AL, CR, and HFD mice. **(C)** ATAC fragment length in all sequenced cells from AL, CR, and HFD mice. **(D)** Transcription start-site (TSS) enrichment in AL, CR, and HFD samples. **(E)** Pie chart showing the breakdown of ATAC peak distribution in types of genomic loci. **(F)** Graph with the number of peaks and RNA molecules with unique molecular identifiers (UMI) in AL, CR, and HFD cells. **(G)** Representative immunohistochemistry (IHC) and confocal microscopy of AL and CR islets after 2 months on diet using anti-Pdx1 and anti-Bmal antibodies. Graph on the right shows the relative frequency of beta cells with high versus low levels of nuclear *Bmal*. **(H-I)**, differentially expressed genes and chromatin accessibility sites in beta cell subpopulations, respectively, of AL, CR, and HFD islets. **(J)** List of genes mapped closest to enriched accessible chromatin sites in different beta cell populations.

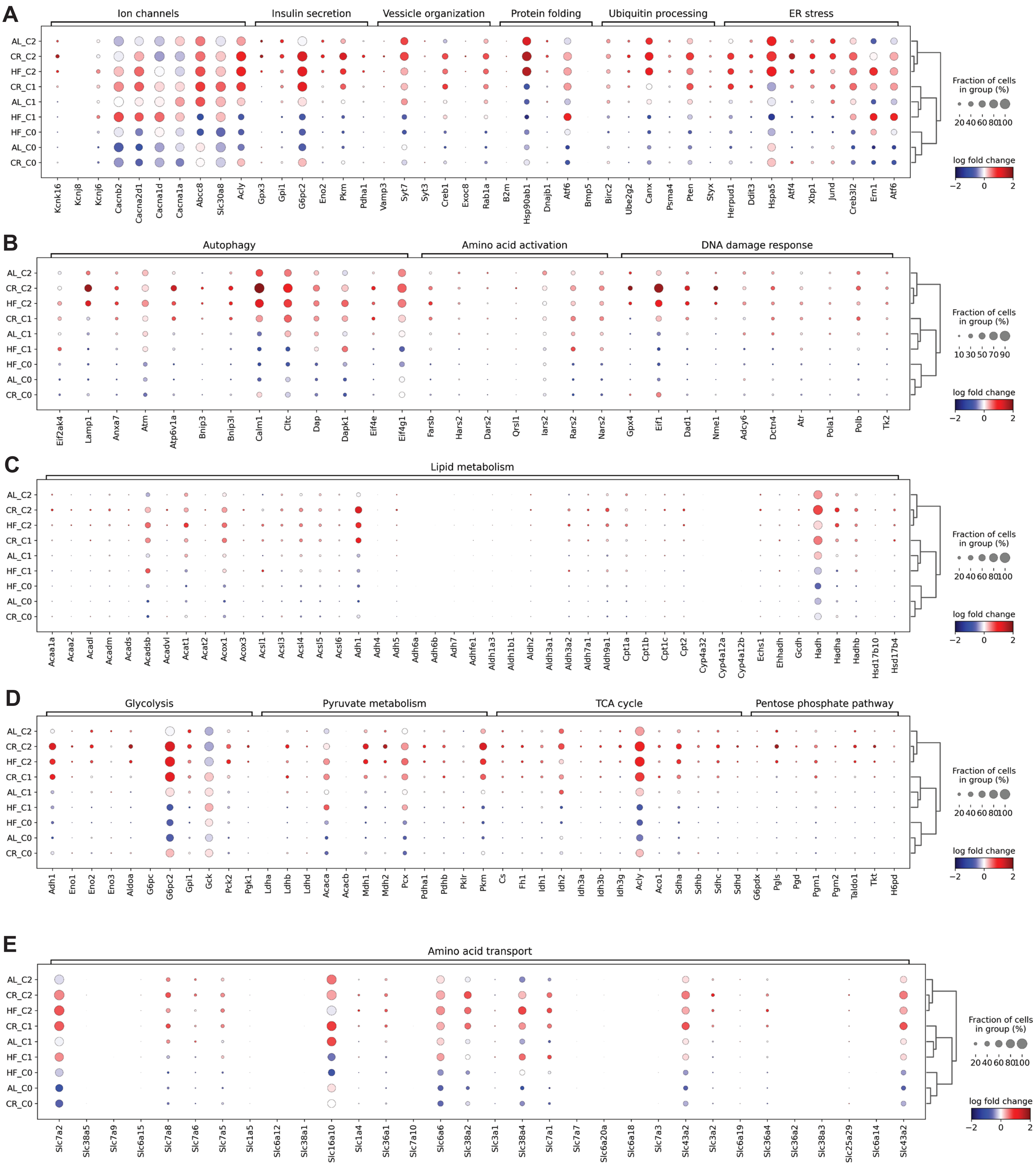
A
Ion channels
Insulin secretion
Vessicle organization
Protein folding
Ubiquitin processing
ER stress
AL_C2
CR_C2
HF_C2
CR_C1
AL_C1
HF_C1
HF_C0
AL_C0
CR_C0
Kcnk16 Kcnj8 Kcnj6 Cacnb2 Cacna2d1 Cacna1d Cacna1a Abcc8 Slc30a8 Acly Gpx3 Gpi1 G6pc2 Eno2 Pkm Pdha1 Vamp3 Syt7 Syt3 Creb1 Exoc8 Rab1a B2m Hsp90ab1 Dnajb1 Atf6 Bmp5 Birc2 Ube2g2 Canx Psma4 Pten Styx Herpud1 Ddit3 Hspa5 Atf4 Xbp1 Jund Creb3l2 Ern1 Atf6
Fraction of cells in group (%)
20 40 60 80 100
log fold change
−2 0 2
B
Autophagy
Amino acid activation
DNA damage response
Eif2ak4 Lamp1 Anxa7 Atm Atp6v1a Bnip3 Bnip3l Calm1 Cltc Dap Dapk1 Eif4e Eif4g1 Farsb Hars2 Dars2 Qrsl1 Iars2 Rars2 Nars2 Gpx4 Eif1 Dad1 Nme1 Adcy6 Dctn4 Atr Pola1 Polb Tk2
10 30 50 70 90
C
Lipid metabolism
Acaa1a Acaa2 Acadl Acadm Acads Acadsb Acadvl Acat1 Acat2 Acox1 Acox3 Acsl1 Acsl3 Acsl4 Acsl5 Acsl6 Adh1 Adh4 Adh5 Adh6a Adh6b Adh7 Adhfe1 Aldh1a3 Aldh1b1 Aldh2 Aldh3a1 Aldh3a2 Aldh7a1 Aldh9a1 Cpt1a Cpt1b Cpt1c Cpt2 Cyp4a32 Cyp4a12a Cyp4a12b Echs1 Ehhadh Gcdh Hadh Hadha Hadhb Hsd17b10 Hsd17b4
D
Glycolysis
Pyruvate metabolism
TCA cycle
Pentose phosphate pathway
Adh1 Eno1 Eno2 Eno3 Aldoa G6pc G6pc2 Gpi1 Gck Pck2 Pgk1 Ldha Ldhb Ldhd Acaca Acacb Mdh1 Mdh2 Pcx Pdha1 Pdhb Pklr Pkm Cs Fh1 Idh1 Idh2 Idh3a Idh3b Idh3g Acly Aco1 Sdha Sdhb Sdhc Sdhd G6pdx Pgls Pgd Pgm1 Pgm2 Taldo1 Tkt H6pd
E
Amino acid transport
Slc7a2 Slc38a5 Slc7a9 Slc6a15 Slc7a8 Slc7a6 Slc7a5 Slc1a5 Slc6a12 Slc38a1 Slc16a10 Slc1a4 Slc36a1 Slc7a10 Slc6a6 Slc38a2 Slc3a1 Slc38a4 Slc7a1 Slc7a7 Slc6a20a Slc6a18 Slc7a3 Slc43a2 Slc3a2 Slc6a19 Slc36a4 Slc36a2 Slc38a3 Slc25a29 Slc6a14 Slc43a2

Supplementary Figure 5. Related to Figure 3. (A-E) Dot plot with hierarchical clustering analysis (HCA) highlighting the expression levels of genes associated with **(A)** ion channels, insulin secretion, vesicle organization, protein folding, ubiquitin processing and endoplasmic reticulum (ER) stress in each beta-cell state; **(B)** autophagy, amino acid activation and DNA damage in each beta-cell state; **(C)** lipid metabolism in each beta-cell state; **(D)** glycolysis, pyruvate metabolism, tricarboxylic acid cycle (TCA) and pentose phosphate pathway, and **(E)** amino acid transport in each beta-cell state.

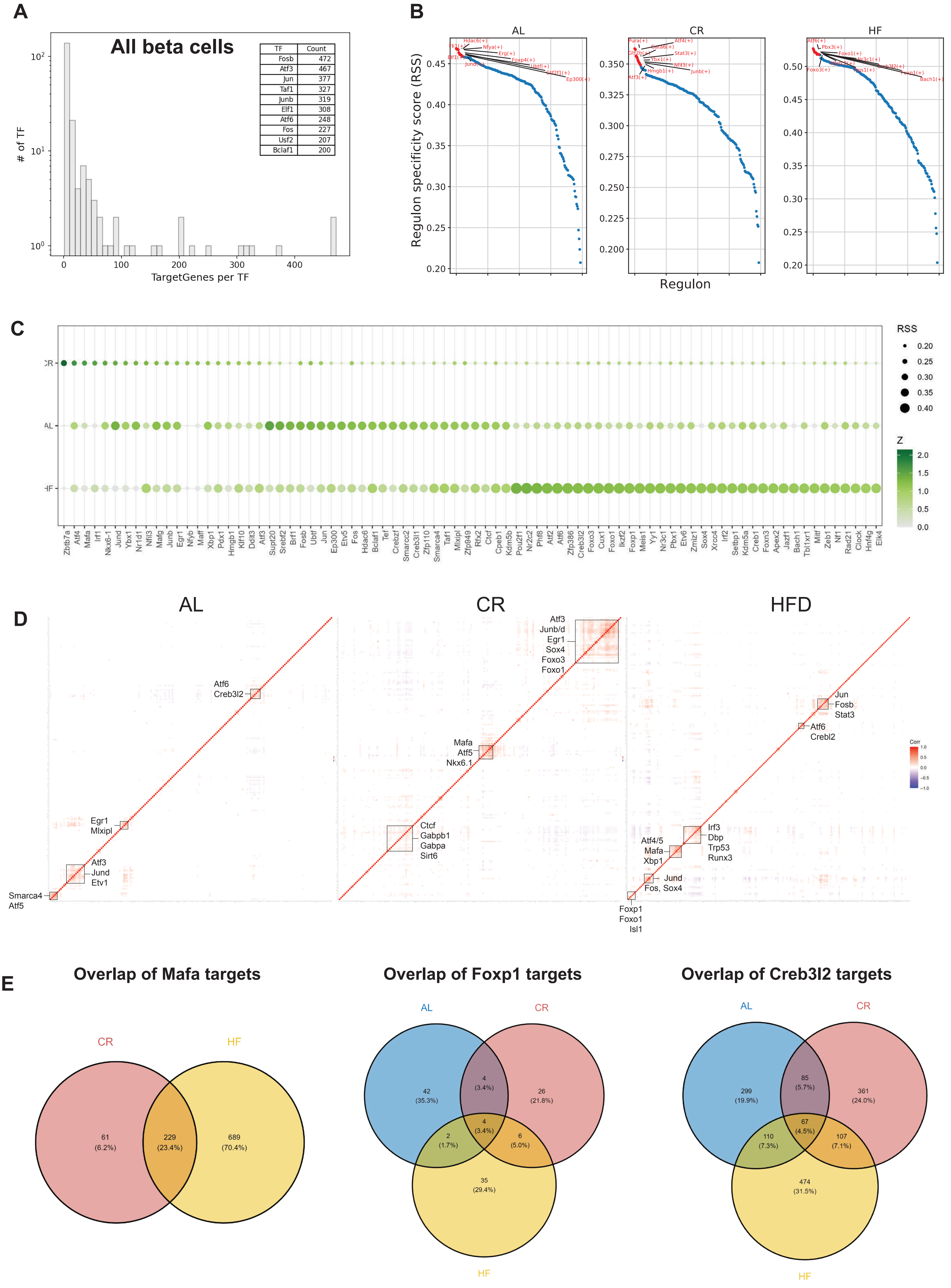
A
All beta cells
TF | Count
Fosb 472
Atf3 467
Jun 377
Taf1 327
Junb 319
Elf1 308
Atf6 248
Fos 227
Usf2 207
Bclaf1 200
# of TF
TargetGenes per TF
B
AL
CR
HF
Regulon specificity score (RSS)
Regulon
C
RSS
Z
CR
AL
HF
Zbtb7a Atf4 Mafa Irf1 Nkx6-1 Jund Ybx1 Nr1d1 Nfil3 Mafg Junb Egr1 Nfyb Maff Xbp1 Pdx1 Hmgb1 Klf10 Ddit3 Atf3 Supt20 Srebf2 Brf1 Fosb Ubtf Jun Ep300 Etv5 Fos Hdac6 Bclaf1 Tef Crebzf Smarcc2 Creb3l1 Zfp110 Smarca4 Taf1 Mlxipl Zfp949 Rfx2 Ctcf Cpeb1 Kdm5b Pou2f1 Nr2c2 Phf8 Atf2 Atf6 Zfp386 Creb3l2 Foxo3 Cux1 Foxo1 Ikzf2 Foxp1 Meis1 Yy1 Nr3c1 Pbx1 Etv6 Zmiz1 Sox4 Xrcc4 Irf2 Setbp1 Kdm5a Creb1 Foxn3 Apex2 Jazf1 Bach1 Tbl1xr1 Mitf Zeb1 Nf1 Rad21 Clock Hnf4g Elk4
D
AL
CR
HFD
Atf6 Creb3l2
Egr1 Mlxipl
Atf3 Jund Etv1
Smarca4 Atf5
Atf3 Junb/d Egr1 Sox4 Foxo3 Foxo1
Mafa Atf5 Nkx6.1
Ctcf Gabpb1 Gabpa Sirt6
Foxp1 Foxo1 Isl1
Jun Fosb Stat3
Atf6 Crebl2
Irf3 Dbp Trp53 Runx3
Atf4/5 Mafa Xbp1
Jund Fos, Sox4
Corr
E
Overlap of Mafa targets
CR
HF
61 (6.2%)
229 (23.4%)
689 (70.4%)
Overlap of Foxp1 targets
AL
CR
HF
42 (35.3%)
4 (3.4%)
26 (21.8%)
4 (3.4%)
2 (1.7%)
6 (5.0%)
35 (29.4%)
Overlap of Creb3l2 targets
AL
CR
HF
299 (19.9%)
85 (5.7%)
361 (24.0%)
67 (4.5%)
110 (7.3%)
107 (7.1%)
474 (31.5%)

Supplementary Figure 6 – Related to Figure 4. (A) Graph with the number of transcription factors (TFs) identified with SCENIC by the number of targets per TF. **(B)** SCENIC regulon specificity score (RSS) for individual TFs in AL, CR, or HFD beta cells. **(C)** RSS and Z-scores for TFs enriched in AL, CR, and HFD beta cells. **(D)** Pearson correlation matrix of TFs identified in mouse beta cells from AL, CR, or HFD mice. Boxes highlight clusters of TFs with high degree of correlation. **(E)** Venn diagrams illustrating the degree of overlap for *Mafa*, *Foxp1*, or *Creb3l2* regulons.

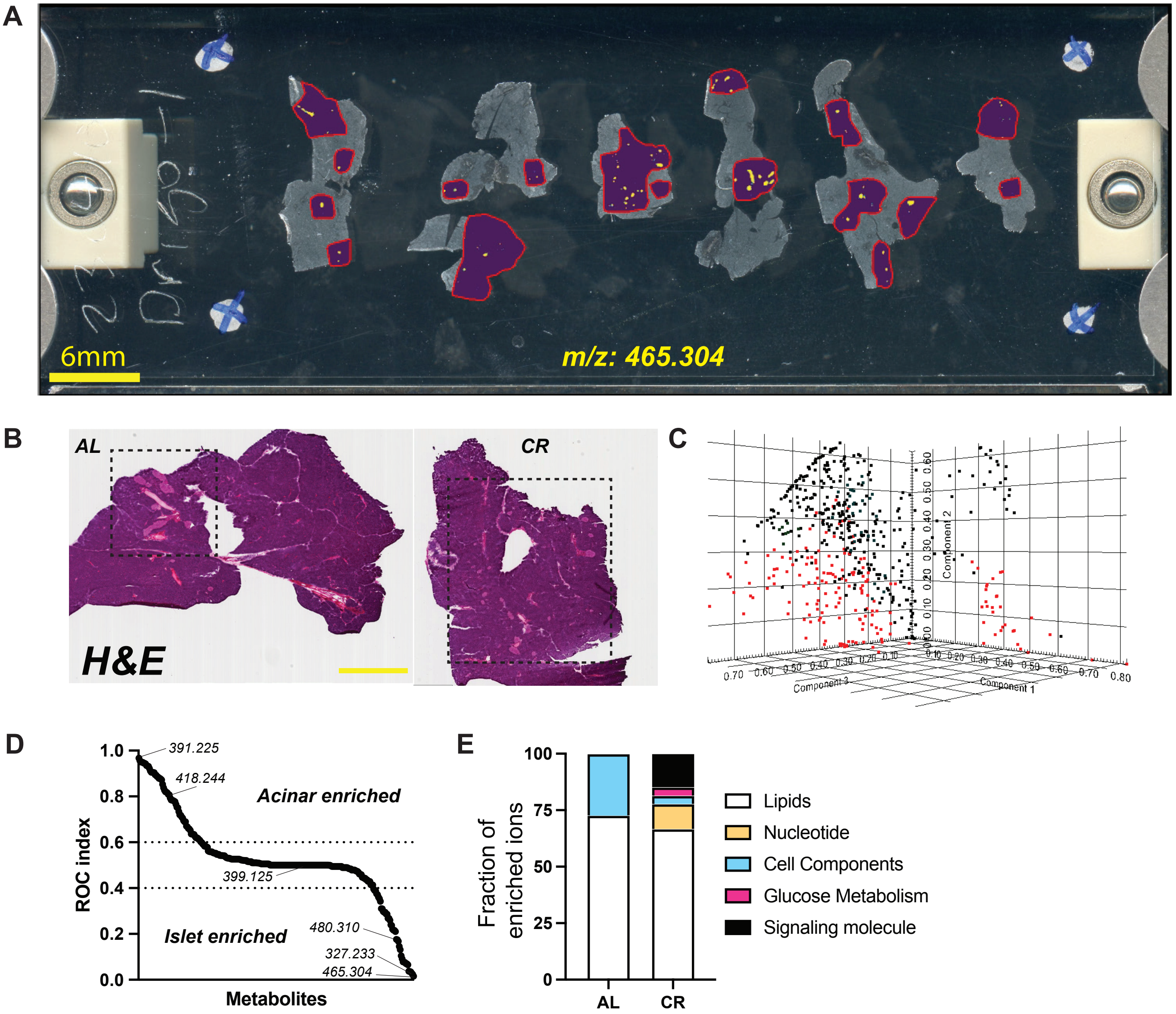
A
6mm
m/z: 465.304
B
AL
CR
H&E
C
Component 2
Component 3
Component 1
D
ROC index
391.225
418.244
Acinar enriched
399.125
Islet enriched
480.310
327.233
465.304
Metabolites
E
Fraction of enriched ions
Lipids
Nucleotide
Cell Components
Glucose Metabolism
Signaling molecule
AL
CR

Supplementary Figure 7 – Related to Figure 5. (A) Overview of MALDI-MS imaging slide with AL and CR pancreas sections. Image is an overlay of brightfield slide imaging annotated with tissue regions of interest (ROIs) used to guide MALDI-MS data acquisition. MALDI-MS data of the islet enriched ion cholesterol sulfate is shown to pinpoint the location of islets *in situ*. **(B)** Representative hematoxylin and eosin (H&E) staining of AL/CR pancreases prepared for MALDI-MS imaging. (C) Three-dimensional graph representing the top 3 components identified using Probabilistic latent semantic analysis (pLSA) analysis with random seed. Individual islets from AL and CR samples are shown in black and red squares, respectively. **(D)** Receiver Operating Characteristic (ROC) analysis of ions enriched in acinar versus islet regions. **(E)** Fraction of metabolites identified against the human metabolome database (HMDB) and their respective molecular classes and distribution in AL or CR datasets. In **(A)**, scale bar = 6mm and in (B), scale bar 3mm.

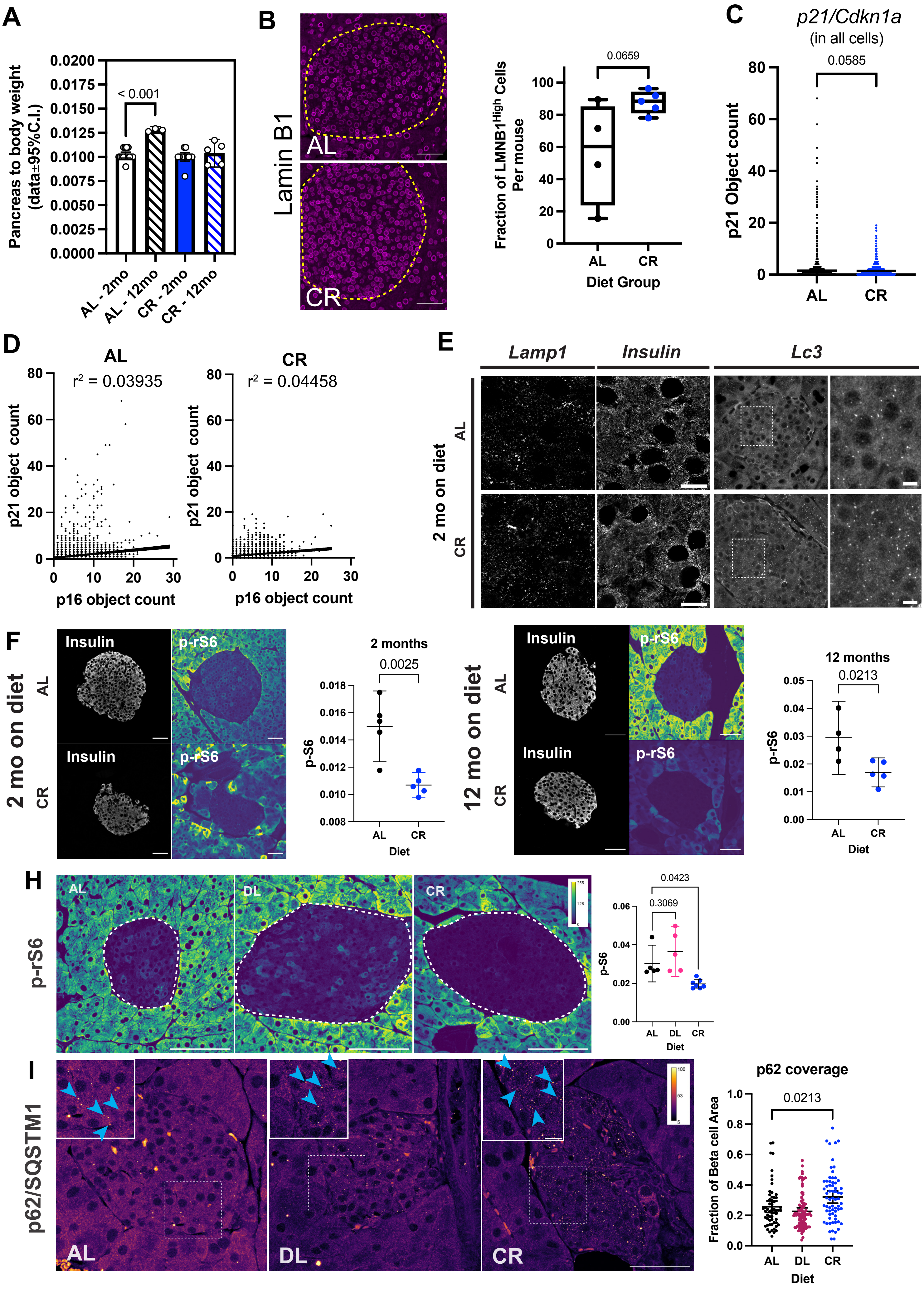
A
Pancreas to body weight (data±95%C.I.)
< 0.001
AL - 2mo
AL - 12mo
CR - 2mo
CR - 12mo
B
Lamin B1
AL
CR
Fraction of LMNB1High Cells Per mouse
0.0659
Diet Group
C
p21/Cdkn1a (in all cells)
0.0585
p21 Object count
D
AL
r^2 = 0.03935
CR
r^2 = 0.04458
p21 object count
p16 object count
E
Lamp1
Insulin
Lc3
2 mo on diet
F
2 mo on diet
Insulin
p-rS6
2 months
0.0025
p-S6
Diet
12 mo on diet
12 months
0.0213
H
p-rS6
AL
DL
CR
0.0423
0.3069
I
p62/SQSTM1
p62 coverage
0.0213
Fraction of Beta cell Area
Diet

Supplementary Figure 8 – Related to Figure 6. **(A)** Relative pancreatic weight in AL and 20% CR mice after 2 or 12 months on diet. **(B)** Representative images of pancreatic islets stained with Lamin B1 from AL and CR male mice after 12 months on diet and the respective quantification of Lamin B1 high cells per mice. Scale bar, 50 microns. **(C)** Quantification of p21 expression *in situ* in pancreatic beta-cells from AL and CR mice after 12 months on diet. **(D)** XY plot showing the correlation between the number of p16 and p21 mRNA spot-object detected in each beta cell from mice kept on AL or CR for 12 months. Solid and dashed lines indicate the linear regression and confidence intervals, respectively. r2 values are shown on top of each graph. **(E)** Representative images of pancreatic islets stained with Lamp1 or Lc3 I-II from AL and CR male mice after 2 months on diet. Scale bar, 10 microns. **(F)** Representative images and quantification of p-rS6 intensity in pancreatic beta-cells after 2 and 12 months on diet. Scale bar, 20 microns. **(H)** Same as in (F), this time in mice fed AL, CR, or DL for 2 months. **(I)** Representative images showing p62 puncta accumulation in mouse beta cells. Quantification is shown on the right. Here, each dot = 1 islet, data pooled from at least n=5 mice per diet group. In (H-I), scale bar 50 microns. Statistical analysis was conducted using one-way ANOVA with Tukey's post-hoc test for multiple comparison (A, H), or unpaired two-tailed Student's t-test (B, C and F). In (A), the asterisks indicate *** p <0.001. In (F-H), each dot = mean from all islets from one individual animal. At least 10-to-20 islets per animal were quantified. All data presented as mean ± 95% C.I.

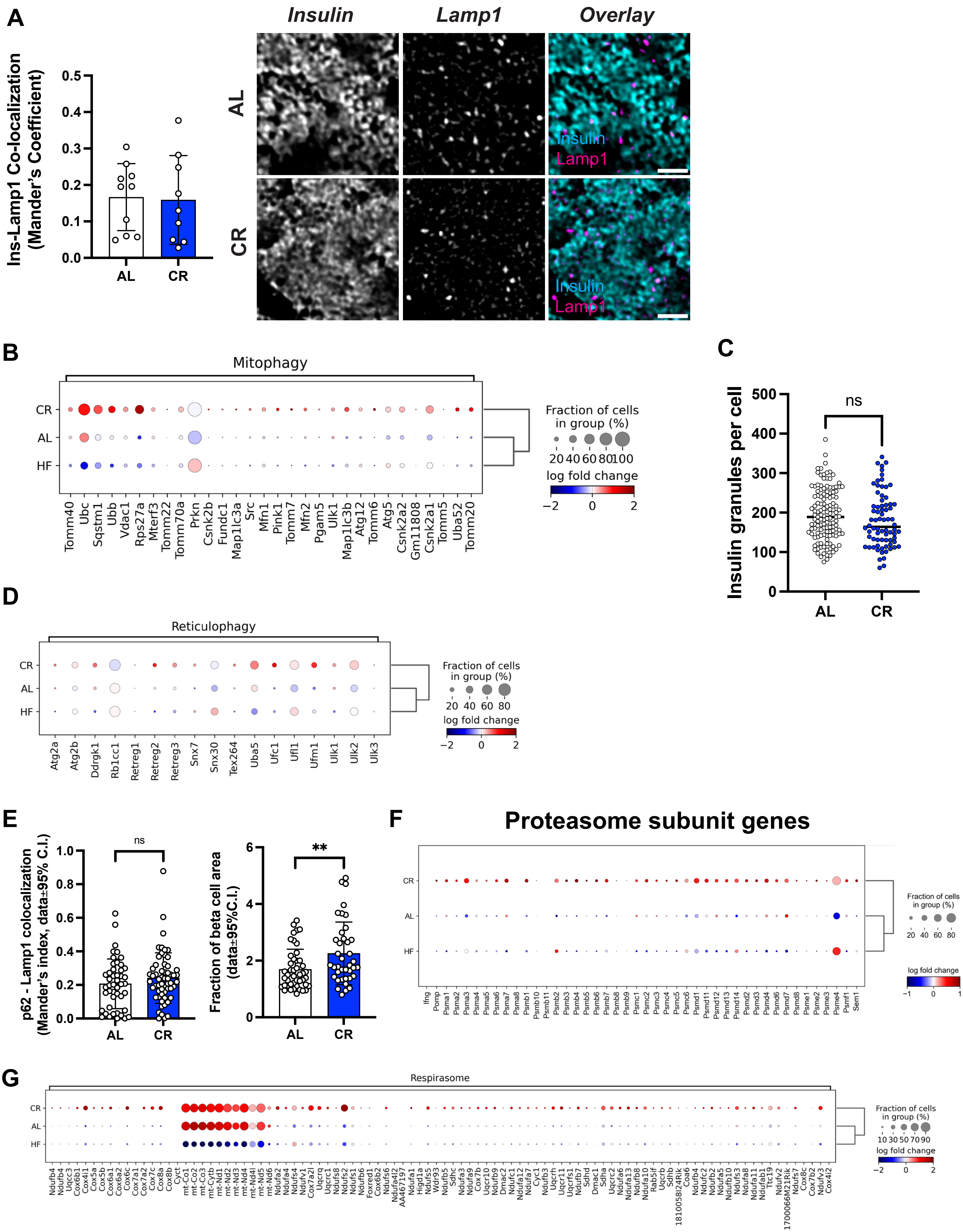
A
Insulin
Lamp1
Overlay
Ins-Lamp1 Co-localization (Mander's Coefficient)
AL
CR
Insulin
Lamp1
B
Mitophagy
CR
AL
HF
Tomm40 Ubc Sqstm1 Ubb Vdac1 Rps27a Mterf3 Tomm22 Tomm70a Prkn Csnk2b Fundc1 Map1lc3a Src Mfn1 Pink1 Tomm7 Mfn2 Pgam5 Ulk1 Map1lc3b Atg12 Tomm6 Atg5 Csnk2a2 Gm11808 Csnk2a1 Tomm5 Uba52 Tomm20
Fraction of cells in group (%)
20 40 60 80 100
log fold change
−2 0 2
C
Insulin granules per cell
ns
AL
CR
D
Reticulophagy
CR
AL
HF
Atg2a Atg2b Ddrgk1 Rb1cc1 Retreg1 Retreg2 Retreg3 Snx7 Snx30 Tex264 Uba5 Ufc1 Ufl1 Ufm1 Ulk1 Ulk2 Ulk3
Fraction of cells in group (%)
20 40 60 80
log fold change
−2 0 2
E
p62 - Lamp1 colocalization (Mander's index, data±95% C.I.)
ns
Fraction of beta cell area (data±95%C.I.)
**
AL
CR
F
Proteasome subunit genes
CR
AL
HF
Fraction of cells in group (%)
20 40 60 80
log fold change
−1 0 1
G
Respirasome
CR
AL
HF
Fraction of cells in group (%)
10 30 50 70 90
log fold change
−2 0 2

Supplementary Figure 9 – Related to Figure 7. **(A)** Co-localization analysis between Insulin and the autophagy marker Lamp1 using pancreatic sections from AL and CR male mice after 2 months on diet. Here, each dot = 1 islet, pooled from n=5 mice per diet group Close up and representative images of beta cells stained with Insulin and Lamp1 are shown on the right. Scale bar, 2 microns. **(B)** Dot plot with hierarchical clustering analysis (HCA) highlighting the expression levels of genes associated with mitophagy. **(C)** Quantification of insulin granules per beta-cells using SEM imaging. **(D)** Same as in **(B)** showing genes involved in the reticulophagy pathway in beta-cells from AL, CR and HFD mice. **(E)** Colocalization of beta cell p62 vesicles with Lamp1 and quantification of beta cell area occupied by p62 in AL or CR mice. In **(F-G),** same as in **(B)** showing genes involved in the proteasome and respirasome pathways, respectively, of AL, CR, or HFD beta cells. In (C and E), each dot = 1 beta-cell, pooled from n=5 mice per diet group. Statistical analysis was conducted using unpaired two-tailed Student's t-test. In (E), the asterisks indicate ** p <0.01. All data presented as mean ± 95% C.I.

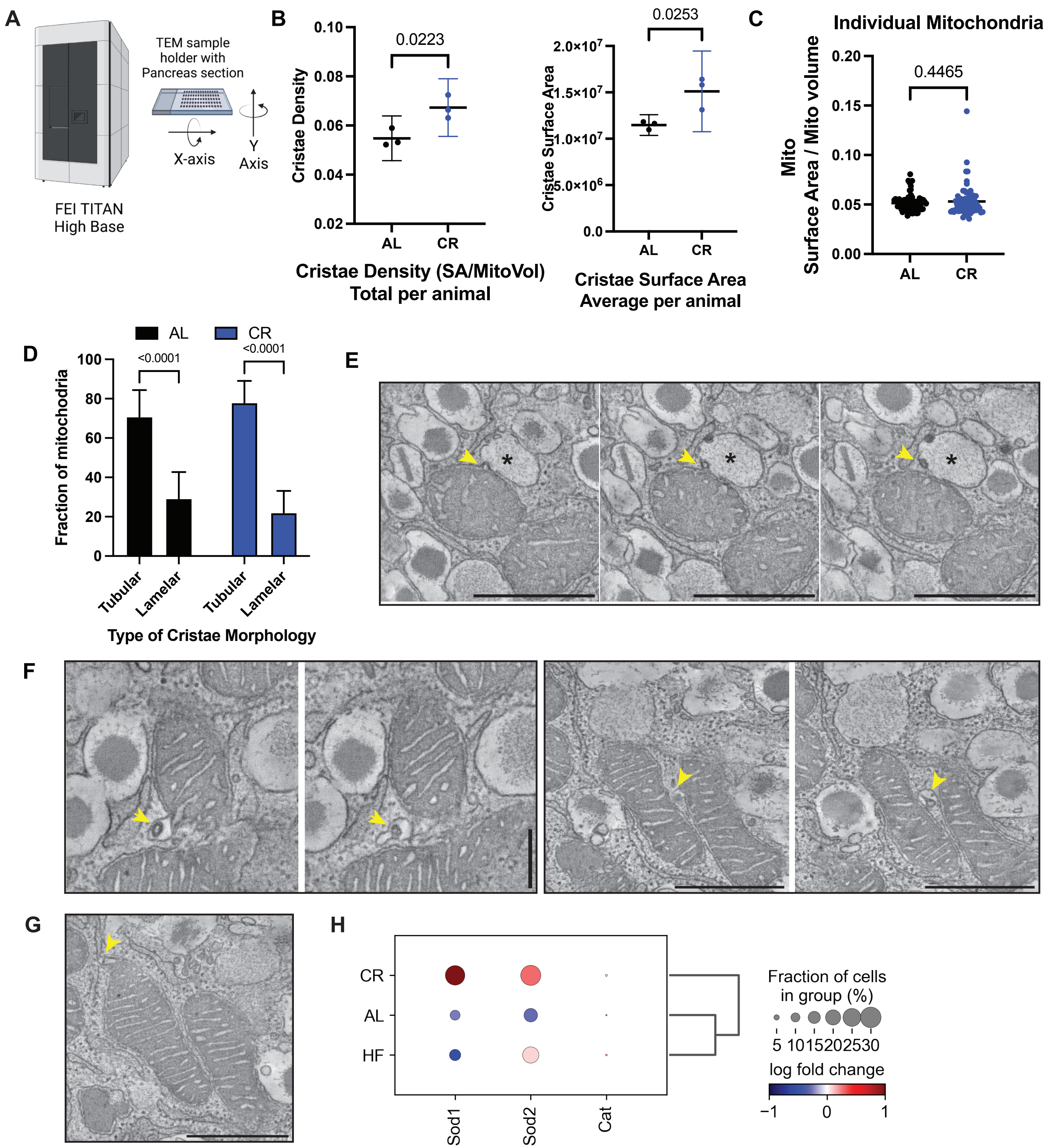
A
TEM sample holder with Pancreas section
X-axis
Y Axis
FEI TITAN High Base
B
0.0223
Cristae Density
AL
CR
Cristae Density (SA/MitoVol) Total per animal
0.0253
Cristae Surface Area
Cristae Surface Area Average per animal
C
Individual Mitochondria
0.4465
Mito Surface Area / Mito volume
D
AL
CR
<0.0001
Fraction of mitochondria
Tubular
Lamelar
Type of Cristae Morphology
E
F
G
H
CR
AL
HF
Sod1
Sod2
Cat
Fraction of cells in group (%)
5 10 15 20 25 30
log fold change
−1
0
1

Supplementary Figure 10 - Related to Figure 7. **(A)** Schematic diagram of eTomo microscopy. **(B)** Average mitochondria cristae density and cristae surface area for each animal analyzed in AL and CR diet groups. **(C)** Mitochondrial surface area to volume ratios in eTomo images. Each dot represents individual mitochondrial objects analyzed. **(D)** Fraction of mitochondria with tubular versus lamellar cristae architecture. **(E-F)** Consecutive z sections showing fusion of a beta cell MDVs with a lysosome. Yellow arrowheads indicate MDVs with multi-membranous structures budding of from the mitochondria outer membrane space and the asterisk marks the lysosome. **(G)** Snapshot of a beta cell MDVs fusing with the ER. In (E-G), yellow arrowheads indicate MDVs locations. **(H)** Dot plot with genes involved in the reactive oxygen species (ROS) pathway in AL, CR, or HFD beta cells. Data pooled from n=3 mice per diet group. Statistical analysis was conducted using unpaired two-tailed Student's t-test. P values are shown. All data presented as mean ± 95% C.I. Panel 10A was created with BioRender.com. and released under a Creative Commons Attribution-Non Commercial NoDerivs 4.0 International license.

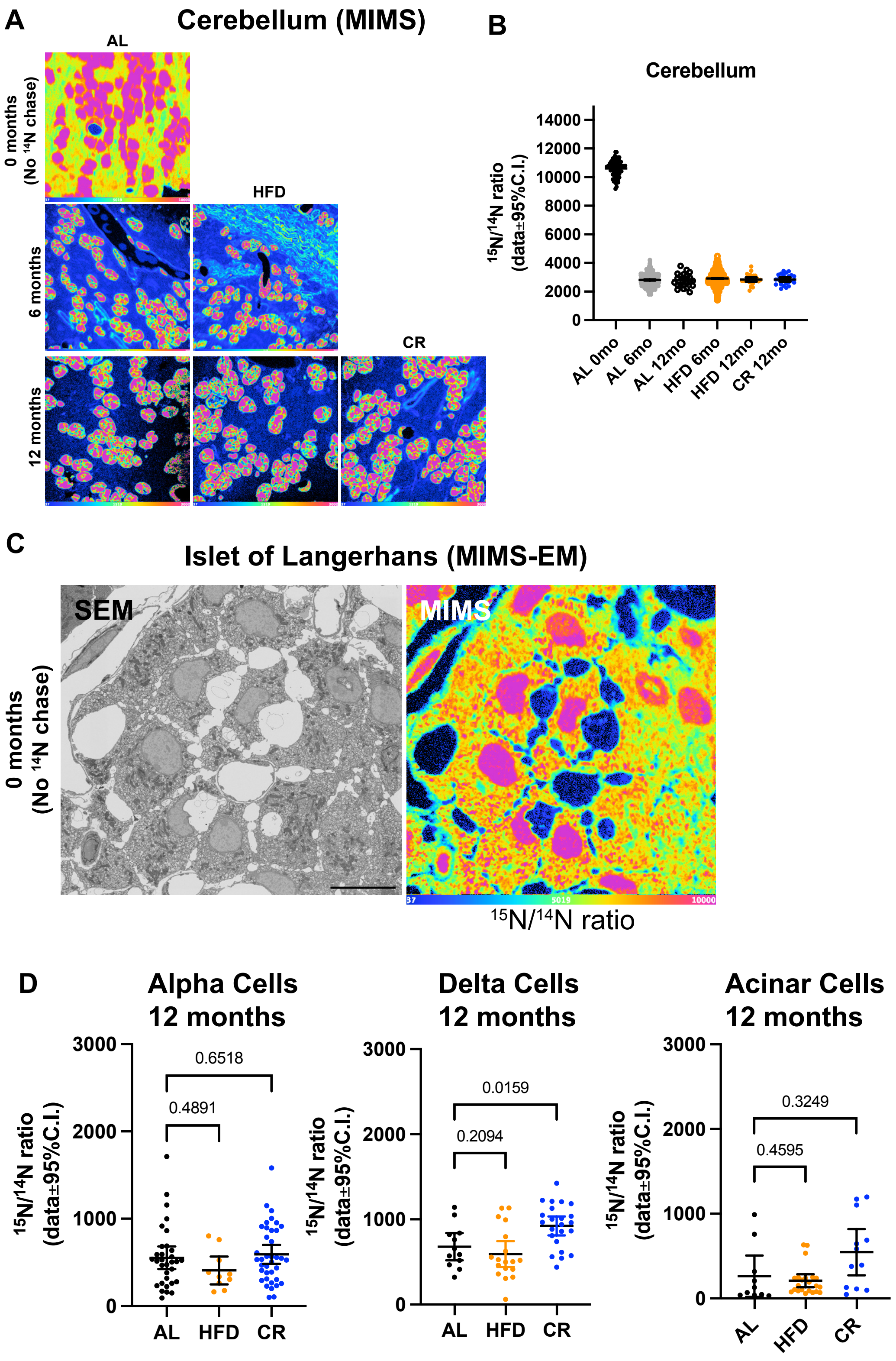
A
Cerebellum (MIMS)
AL
HFD
CR
0 months (No 14N chase)
6 months
12 months
B
Cerebellum
15N/14N ratio (data±95%C.I.)
14000
12000
10000
8000
6000
4000
2000
0
AL 0mo
AL 6mo
AL 12mo
HFD 6mo
HFD 12mo
CR 12mo
C
Islet of Langerhans (MIMS-EM)
SEM
MIMS
0 months (No 14N chase)
37
5019
10000
15N/14N ratio
D
Alpha Cells 12 months
Delta Cells 12 months
Acinar Cells 12 months
15N/14N ratio (data±95%C.I.)
3000
2000
1000
0
0.6518
0.4891
0.0159
0.2094
0.3249
0.4595
AL
HFD
CR

Supplementary Figure 11 - Related to Figure 8. **(A)** MIMS imaging and $^{15}N/^{14}N$ ratiometric maps of granule neurons in the cerebellum of ^{15}N-labelled mice at day 0 or after 6 or 12 months of chase in AL, HFD, or CR diet groups. **(B)** $^{15}N/^{14}N$ values from granule neurons from mice kept on AL, HFD, or CR for 6 or 12 months. Each dot represents a single nucleus. **(C)** SEM and MIMS imaging with $^{15}N/^{14}N$ ratiometric maps of islet cells in the pancreas from a ^{15}N-labelled mouse at day 0. **(D)** $^{15}N/^{14}N$ values in the nucleus of alpha cells, delta cells, and acinar cells from the pancreas of mice kept on AL, HFD, or CR for 12 months. Each dot represents a single cell nucleus. In (C), scale bar = 10 microns. Statistical analysis was conducted using one-way ANOVA with Tukey's post-hoc test. P values are shown. All data presented as mean ± 95% C.I.
