# Supplementary material for: Calorie restriction increases insulin sensitivity to promote beta cell homeostasis and longevity in mice
Source: Nat Commun. 2024 Oct 21;15:9063. doi: 10.1038/s41467-024-53127-2 (PMC11493975; doi:10.1038/s41467-024-53127-2)
Supplement: Supplementary file 3 — Description of Additional Supplementary Files [file 41467_2024_53127_MOESM3_ESM.docx]

**Description of Additional Supplementary Files**

**Supplementary Data 1.** List of differentially expressed of genes and pathway enrichment analysis in CR-HF vs AL-HF islets.

**Supplementary Data 2.** List of differentially expressed of genes (DEG) in AL, CR, or HFD beta cells.

**Supplementary Data 3.** List of differentially regulated transcription factor motifs in AL, CR, or HFD beta cells.

**Supplementary Data 4.** List of differentially expressed of genes (DEG) in the identified beta cell states of AL, CR, or HFD beta cells

**Supplementary Data 5.** List of transcription factors identified using SCENIC analysis of AL, CR, or HFD beta cell transcriptomics.

**Supplementary Data 6.** Detailed list of genes associated with regulons for Mafa, Foxp1, or Crebl2 in AL, HFD, and CR beta cells.

**Supplementary Data 7.** Full list of genes associated with all identified regulons in AL, HFD, and CR beta cells.

**Supplementary Data 8.** ROC analysis of metabolite ions identified in islet or acinar compartments of AL or CR pancreases.

**Supplementary Data 9.** Complete list of possible metabolites associated with all possible molecular identifiers.

**Supplementary Video 1.** Movie of a representative beta cell eTomo acquisition field of view from an AL mouse islet.
